# Three Eminent Physicians

**Published:** 1886-06

**Authors:** 


**Three Eminent Physicians.**—As the celebrated French physician Desmoulins lay on his death-bed he was visited and almost constantly surrounded by the most distinguished medical men of Paris, as well as other prominent citizens of the French metropolis. Great were the lamentations of all at the loss about to be sustained by the profession in the death of one they regarded as its brightest ornament; but Desmoulins spoke cheerfully to his fellow-practitioners, assuring them that he had left behind three physicians much greater than himself. Each of the doctors, hoping that his own name would be called, inquired anxiously who was sufficiently illustrious to surpass the immortal Desmoulins. With great distinctness the dying man answered, "They are Water, Exercise and Diet. Call in the service of the first freely, of the second regularly, and of the third moderately. Follow this advice and you may dispense with my aid. Living, I could do nothing without them, and dying I shall not be missed if you make friends with these my faithful coadjutors."

